# Ferroptosis Signaling and Regulators in Atherosclerosis

**DOI:** 10.3389/fcell.2021.809457

**Published:** 2021-12-16

**Authors:** Yuqin Wang, Yajie Zhao, Ting Ye, Liming Yang, Yanna Shen, Hong Li

**Affiliations:** ^1^ Department of Pathophysiology, School of Basic Medical Sciences, Harbin Medical University, Harbin, China; ^2^ Department of Pathophysiology, Harbin Medical University-Daqing, Daqing, China; ^3^ School of Medical Laboratory, Tianjin Medical University, Tianjin, China

**Keywords:** ferroptosis, atherosclerosis, Nrf2, p53, Hippo

## Abstract

Atherosclerosis (AS) is a major cause of cardiovascular diseases such as coronary heart disease, heart failure and stroke. Abnormal lipid metabolism, oxidative stress and inflammation are the main features of AS. Ferroptosis is an iron-driven programmed cell death characterized by lipid peroxidation, which have been proved to participate in the development and progression of AS by different signal pathways. NRF2-Keap1 pathway decreases ferroptosis associated with AS by maintaining cellular iron homeostasis, increasing the production glutathione, GPX4 and NADPH. The p53 plays different roles in ferroptosis at different stages of AS in a transcription-dependent and transcription- independent manner. The Hippo pathway is involved in progression of AS, which has been proved the activation of ferroptosis. Other transcription factors, such as ATF3, ATF4, STAT3, also involved in the occurrence of ferroptosis and AS. Certain proteins or enzymes also have a regulatory role in AS and ferroptosis. In this paper, we review the mechanism of ferroptosis and its important role in AS in an attempt to find a new relationship between ferroptosis and AS and provide new ideas for the future treatment of AS.

## 1. Introduction

The main lesion of AS is lipid deposition in the arterial wall, accompanied by proliferation of smooth muscle cells and fibrous matrix, which gradually forms atherosclerotic plaques (Zhu et al., 2018). AS is the pathological basis of cardiovascular disease, while rupture of unstable atherosclerotic plaques, platelet aggregation and thrombosis can lead to narrowing or occlusion of blood vessels, resulting in acute cardiovascular disease (Li and Chen, 2005; Kanter et al., 2012; Zhu et al., 2018). Inflammation plays an important role in all stages of the atherosclerotic process, especially involved in the formation of unstable plaques (Zhu et al., 2018).

Ferroptosis is a regulated cell death dependent on reactive oxygen species (ROS) production and iron overload (Wang et al., 2020a). The basic mechanism of ferroptosis is the interaction of intracellular free iron with hydrogen peroxide via the Fenton reaction, leading to the depletion of plasma membrane polyunsaturated fatty acids (PUFAs) (Stockwell et al., 2017). Ferroptosis is regulated by a variety of cellular metabolic pathways, including redox homeostasis, iron handling, mitochondrial activity and the metabolism of amino acids, lipids and sugars. Metabolic processes that affect cellular susceptibility to ferroptosis include the sulfhydryl-dependent redox system and the mevalonate pathway, while the Cysteine/GSH/GPX4 axis, the NAD(P)H/FSP1/CoQ10 system, and the GCH1/BH4/DHFR system inhibit ferroptosis (Zheng and Conrad, 2020). The transcription factors (such as p53, Nrf2, ATF3, ATF4, YAP1, HIF1a, EPAS1/HIF2A, BACH1, TFEB, Jun, HIC1 and HNF4a) play multiple roles in the regulation of ferroptosis through transcription-dependent or non-transcriptional mechanisms (Dai et al., 2020). In this review, we summarize the signaling pathways of ferroptosis associated with AS in order to provide new ideas for the prevention and treatment of AS.

## 2. Ferroptosis

### 2.1 Lipid Peroxidation and Ferroptosis

**FIGURE 1:**
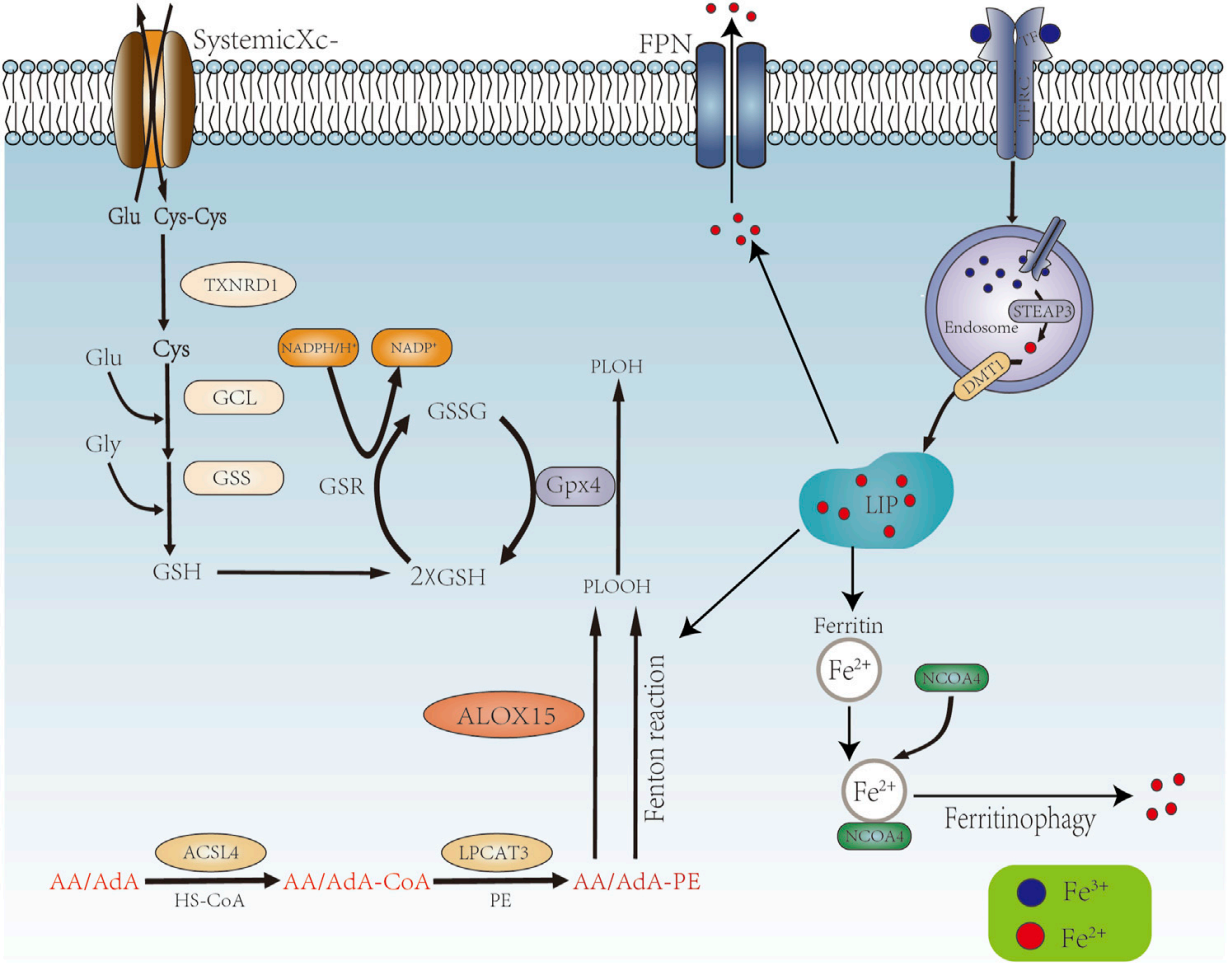
Mechanism of ferroptosis occurrence. AA/AdA is activated by ACSL4 to become AA/AdA-CoA, then AA/AdA-CoA is esterified by LPCAT3 to AA/AdA-PE, AA/AdA-PE generates PLOOH through the Fenton reaction and the enzymatic reaction of ALOX15. Systemic Xc- promotes the synthesis of GSH, which together with GPX4 converts PLOOH to PLOH. Extracellular TF binds to Fe^3+^ and translocated into the cell via TFR1. The cell contains Fe3+, TF and TFR1 into the endosome by endocytosis. STEAP3 reduces Fe^3+^ to Fe^2+^, which is transported to the cytoplasm via DMT1 to form LIP. Fe^2+^ in LIP promote ferroptosis by Fenton reaction. Ferritin reduces Fe^2+^ is decreased by storing of Ferritin and the excrete via FPN. Ferritin also binds to NCOA4 to release Fe^2+^ via ferritinphagy.

Lipid peroxidation, a process of oxidative degradation of lipids, plays a key role in driving ferroptosis. The phospholipid acyl chain remodeling pathway (Lands’ cycle) is critical for achieving ferroptosis. Upon entry of extracellular free diffusible AA/AdA into the cell, acyl-CoA synthetase long chain family member 4 (ACSL4) can activate AA/Ada to generate AA/AdA- CoA, which can then be esterified to AA/AdA-PE by LPCAT3 (lysophosphatidylcholine acyltransferase 3) (Latunde-Dada, 2017). AA/AdA-PEs are the main target molecules for lipid peroxidation and are localized to the cytoplasmic face of the membrane during ferroptosis (Doll and Conrad, 2017). AA/AdA-PE undergoes iron-dependent lipid autoxidation/peroxidation via the Fenton reaction or enzymatic catalysis (e.g., Alox15) to produce phospholipid hydroperoxides (PLOOH) (Doll and Conrad, 2017), which ultimately leads to the formation of a large number of byproducts, including lipid peroxides (such as 4-hydroxynonenal and malondialdehyde) and breakdown products of oxidized and modified proteins, giving rise to the disruption of membrane integrity and ferroptosis (Jiang et al., 2021). As an amino acid reverse transporter protein distributed in the phospholipid bilayer consisting of two subunits, SLC7A11 and SLC3A2, system Xc^−^ exchanges cysteine and glutamate intracellularly and extracellularly in a 1:1 ratio. Cysteine is involved in the synthesis of GSH and the function of GPX4 (glutathione peroxidase 4) (Li et al., 2020) (Figure 1).

There are currently two explains about phenotypic features of ferroptosis observed *in vitro* experiments. One possibility is that the accumulation of PLOOH and breakdown products in the membrane during lipid peroxidation alters the properties of the membrane, leading to the formation of hydrophilic pores and altered permeability of water and other solutes through the cell membrane. Another possibility is that the formation of lipophilic and electrophilic substances during lipid peroxidation act as specific signaling molecules that mediate membrane permeability by modifying downstream molecules (e.g., membrane proteins) (Doll and Conrad, 2017).

### 2.2 Iron Homeostasis and Ferroptosis

The regulation of iron homeostasis is particularly important for maintaining overall redox balance. On the one hand, iron is an essential element for physiological processes such as oxygen transport or energy production. On the other hand, overloaded iron acts as a catalyst for redox reactions, which can lead to oxidative stress-induced cytotoxicity (Galaris and Pantopoulos, 2008; Klóska et al., 2019). The bioavailability of intracellular iron is regulated by its import, storage and export (Klóska et al., 2019). Iron is present in two forms, Fe^3+^ and Fe^2+^. Fe^2+^ can generate ROS via the Fenton reaction, leading to the accumulation of lipid peroxides, which is an important factor in the onset of ferroptosis. Extracellular Fe^3+^ can be bound to transferrin (TF) and transported into the cell via Trans-ferrin Receptor 1 (TFR1) on the cell membrane.

Fe^3+^, TF and TFR1 are incorporated into endosomes by endocytosis, while the binding of TF and TFR1 and the binding of iron and TF are solubilized in an acidic environment. Fe^3+^ is reduced to Fe^2+^ in the endosome by STEAP3 (iron reductase), which then mediates the release of Fe^2+^ from the endosome into the cytoplasm via Divalent metal transporter 1 (DMT1/SLC11A2) to form LIP. Cytoplasmic Fe^2+^ can be reduced by ferritin and stored as Fe^3+^, and Fe^2+^ can be transported out of the cell by ferroportin (FPN1/SLC40A1) (Anandhan et al., 2020). Ferritin is an important negative regulator of ferroptosis, consisting of the ferritin heavy chain (FTH1) and the ferritin light chain (FTL). FTH1 has ferrous oxidase activity and oxidizes Fe^2+^ to Fe^3+^, which is then stored in ferritin (Finazzi and Arosio, 2014) (Figure 1).

Ferritinophagy, the autophagic degradation process of the iron storage protein ferritin, is essential for the regulation of cellular iron levels, and requires the co-regulation of NCOA4 (nuclear receptor coactivator 4) and autophagy. Knockout of NCOA4 inhibited erastin induced ferroptosis, while overexpression significantly enhanced intracellular iron levels promoting ferroptosis (Hou et al., 2016). NCOA4 binds to FTH1, autophagic vesicles and autolysosomes, which induce the releases of free iron (Mancias et al., 2014). Depletion or inhibition of NCOA4 or ATG proteins (e.g., ATG3, ATG5, ATG7 and ATG13) inhibits ferritin degradation, thereby reducing free iron levels and limiting ferroptosis (Hou et al., 2016; Gao et al., 2016; Liu et al., 2020) (Figure 1). Lipid peroxides can be produced by three pathways: iron-catalyzed lipid autoxidation, esterification and oxidation of polyunsaturated fatty acids (PUFAs), and the production of lipid ROS associated with the Fenton reaction, all of which require iron (Ying and Padanilam, 2016; Lei et al., 2019; Su et al., 2019; Li et al., 2020).

## 3. Atherosclerosis

AS is characteristic by disorders of lipid metabolism, smooth muscle proliferation, endothelial dysfunction, apoptosis, necrosis, inflammation and the formation of foamy cells and lipid plaques (Wu et al., 2017). AS-associated inflammation is mediated by pro-inflammatory cytokines, inflammatory signaling pathways, bioactive lipids and adhesion molecules (Zhu et al., 2018). The main cells involved in AS are endothelial cells, smooth muscle cells and macrophages, etc. The damage and dysfunction of Endothelial cells is the initiating links in AS, and cholesterol-rich low-density lipoprotein (LDL)is deposited in the inner membrane of the vascular wall of endothelial cells, where it is oxidized to oxidized LDL during increased oxidative stress, which can be recognized by macrophages and other immune cells and destroy endothelial cells (Rafieian-Kopaei et al., 2014; Jinnouchi et al., 2020). The damaged endothelium is activated and expresses cytokines, chemokines and adhesion molecules that attract circulating monocytes to the atherosclerotic lesions and adhere to the vessel wal (Marchio et al., 2019). The main immune cells in atherosclerotic lesions are macrophages, which are divided into the pro-inflammatory macrophage phenotype M1 and the anti-inflammatory macrophage phenotype M2 (Tabas and Bornfeldt, 2016). Scavenger receptors (SR) on the macrophage take part in the lipids dynamic balance dependent on a number of enzymes, such as cholesterol acyltransferase-1 (ACAT1) and the cholesterol efflux transporters ABCA1 and ABCG1 (Linton et al., 2016). Reduced phagocytosis of lipids lead to the conversion of macrophages into foam cells, which induce the formation of plaques. Macrophage-derived foam cell-dominated atherosclerotic plaques are less stable and more likely to rupture.

## 4.Signaling Pathways of Ferroptosis Associated With Atherosclerosis

### 4.1 NRF2-Keap1 Pathway

**FIGURE 2:**
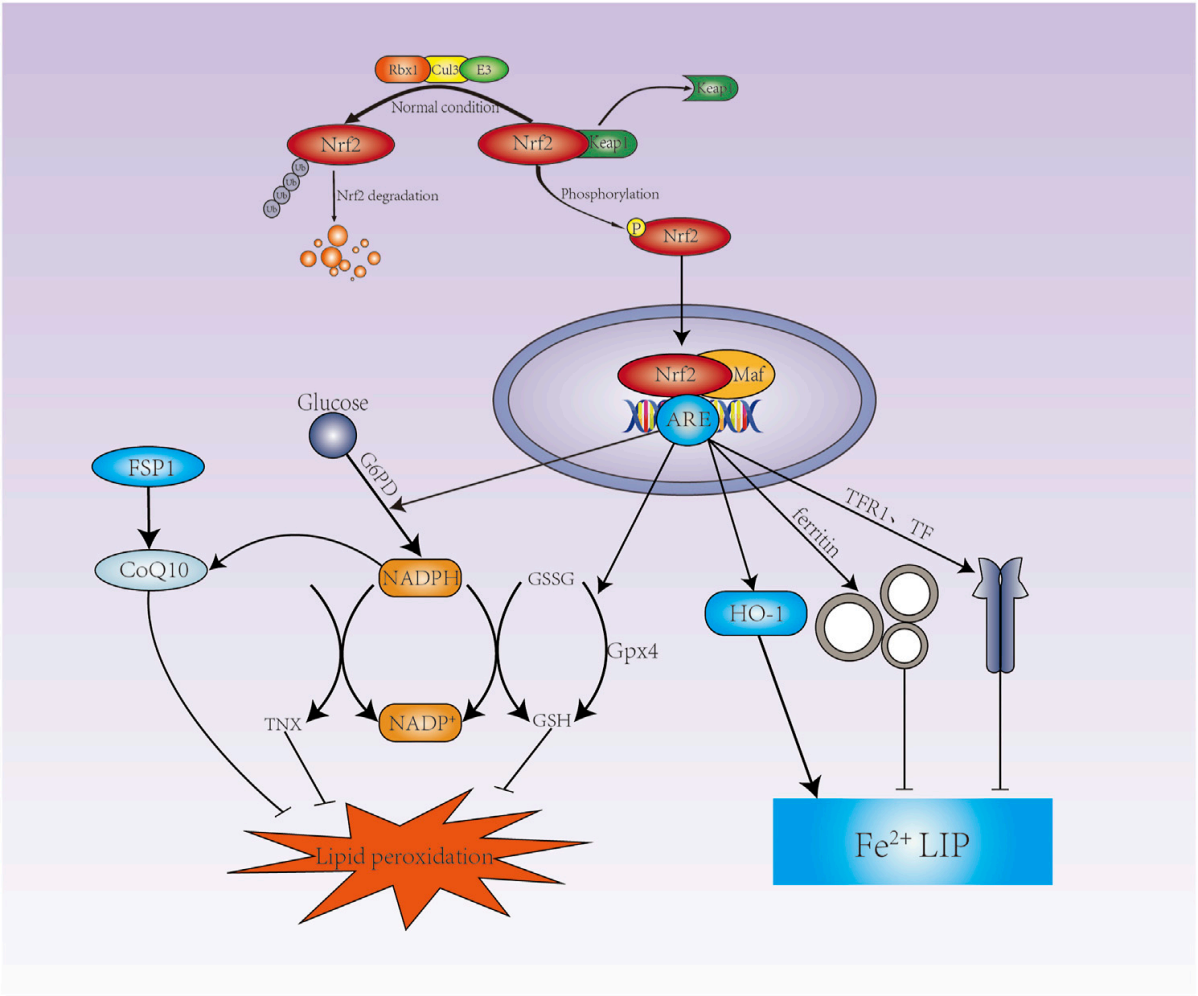
NRF2 is involved in the regulation of ferroptosis. Under normal conditions, NRF2 is degraded by the Keap1-CUL3-RBX1 E3 ubiquitin ligase complex-targeted proteasome. Under oxidative stress conditions, NRF2 is no longer degraded, thus allowing nuclear translocation and binding to the ARE. NRF2 promotes G6PD expression to increases NADPH via the pentose phosphate pathway, GSH and TXN production is dependent on NADPH, which promotes CoQ10 production. NRF2 promotes the expression of SLC7A11, GCLC, GCLM, GSS to increase GSH synthesis, and NRF2 also promotes the expression of GPX4. GPX4 converts GSH to GSSG to reduce lipid hydrogen peroxide. NRF2 can promote the expression of ferritin and FPN to store Fe^2+^ and excrete Fe^2+^ respectively, and reduce Fe^2+^ in LIP; Nrf2 also promote the expression of HO-1.

Activated NRF2 improves the oxidative stress state of the body and promotes cell survival. Under non-stress conditions, NRF2 is ubiquitinated by the Keap1-CUL3 ubiquitin E3 ligase complex to mark it for degradation by the proteasome. However, redox interference leads to its functional inactivation by modifies of Keap1 when exposed to electrophilic substances or reactive oxygen species. Keap1-CUL3 ubiquitin E3 ligase activity is reduced, which promote the stabilization of NRF2. Stabilized and accumulated NRF2 translocate to the nucleus, forms a heterodimer with a small muscle tendon fibrosarcoma (Smaf), then initiates transcription of genes containing antioxidant response elements (ARE) and activates a range of cytoprotective genes. NRF2 has been shown to regulate the activity of ferroptosis and lipid peroxidation related proteins by three main pathways, iron/metal metabolism, intermediate metabolism and GSH synthesis/metabolism (Dodson et al., 2019) (Figure 2).

#### 4.1.1 NRF2 Maintains Iron Homeostasis

Iron can also be released during heme degradation by HO-1 (heme oxygenase), a stress-inducible enzyme encoded by the Hmox1 gene. HO-1 metabolite performs an essential physiological function in the vascular system (Loboda et al., 2008). As a Metabolic product, biliverdin is rapidly reduced to bilirubin by biliverdin reductase, which is expressed in two forms: BVRa (predominant in adults and encoded by BLVRA) and BVRb (encoded by BLVRb and predominant in fetal life) (Baranano et al., 2002). Knockdown of BVR induced an increase in NRF2 transcription factor activity and HO-1 levels, accompanied by decrease of cellular heme content and increase of iron content (Klóska et al., 2019). The binding of NRF2 to the ABCB6 promoter region was first identified in airway epithelial cells of smokers with tissue specificity in the regulation of ABCB6 by NRF2 (Hübner et al., 2009; Campbell et al., 2013; Kerins and Ooi, 2018). Iron is transported from the cytoplasm to the mitochondria via mitoferrin 1 (MFRN1), where iron-containing porphyrins are synthesized to decrease iron ions. Up-regulation of ABCB6 promotes iron depletion by exporting iron-containing porphyrins, thereby inhibiting ferroptosis (Zhang et al., 2020). The lack of ABCB6 in bone marrow cells leads to increased oxidative stress and subsequent release of platelets. Subsequent thrombocytosis increases arterial deposition of the powerful myeloid cell-attracting chemokine CCL5 (RANTES), which ultimately promotes the development of atherosclerotic lesions (Murphy et al., 2014). In addition to ABCB6, ferrous iron chelatase (FECH) is located in the mitochondria and involved in the biosynthesis of hemoglobin. FECH inserts ferrous iron into protoporphyrin IX to produce hemoglobin cofactor (Khan and Quigley, 2011). NRF2 regulates heme synthesis via ABCB6, which is responsible for transporting porphyrins from the cytosol to the mitochondrial membrane gap (Kerins and Ooi, 2018).

NRF2 regulates intracellular iron homeostasis by regulating the expression of Tfr1, FTH1, FTL and FPN. It was found that p62 interacting with Keap1, promotes the translocation of NRF2 into the nucleus, up-regulate the expression of FTH, FPN and HO-1, which protects cells from ferroptosis (Zhao et al., 2021). TfR1 was found to be highly expressed in macrophage-derived foam cells and smooth muscle cells in human carotid plaque (Li et al., 2008). The expression of TfR1 was positively correlated with macrophage infiltration, ectopic lysosomal histone lyase L and ferritin expression (Li et al., 2008). Ox-LDL and pro-inflammatory cytokines promote macrophage iron retention and lipid accumulation, mainly due to NRF2-mediated increased expression of Hmox1 and FPN, a specific cellular phenotype that may be associated with the development of atherosclerotic lesions and plaques instability (Marques et al., 2016).

#### 4.1.2 NRF2 Increases the Expression of GSH and GPX4

NRF2 can regulate intracellular redox homeostasis through genes encoding GSH synthesis proteins, including SLC7A11, TXNRD1, GCLC, GCLM, and GSS (Song and Long, 2020). SLC7A11 is an essential subunit of system Xc- that regulates the reverse transport of glutamate and cystine. In contrast, NRF2 promotes the expression of SLC7A11 (Carpi-Santos and Calaza, 2018). Overexpression of NRF2 or knockdown of Keap1 increases SLC7A11 expression, whereas inhibition of expression of NRF2 or over-expression of Keap1 over-expression decreases SLC7A11 levels (Fan et al., 2017; Shin et al., 2017). It was found that inhibition of system Xc- induced GSH depletion increased the expression of TXNRD1, but not TXNRD2, compensated for the lack of GSH (Mandal et al., 2010). The synthesis of GSH is divided into two steps. The first step is catalyzed by GCL (glutamate-cysteine ligase), which consists of GCLC (GCL-catalytic subunit) and GCLM (GCL-modifier subunit), and cysteine combine with glutamate to produce γ-glutamylcysteine. The second step is catalyzed by GSS (Glutathione synthetase), which adds glycine to γ-glutamylcysteine to form γ-glutamylcysteine or GSH (Kalinina and Gavriliuk, 2020). GPX4 converts GSH to GSSG (oxidized glutathione) to reduce lipid hydrogen peroxide (Gaschler et al., 2018) (Figure 2).

Depletion of the antioxidant GSH or ox-LDL lead to the accumulation of intracellular lipid peroxide (Dixon et al., 2012; Kattoor et al., 2017; Ouyang et al., 2021). Over-expression of GPX4 in ApoE^−/−^ mice attenuates the upregulation of endothelial cell adhesion molecule and monocyte-endothelial cell adhesion, thereby inhibiting the development of AS (Guo et al., 2008). Butyric acid treatment of VSMC not only up-regulates the expression of GPX4 but also enhances the catalytic activity of GPX4, which in turn inhibits VSMC proliferation and enhances arterial protective effect (Mathew et al., 2014). GPX4 also down-regulates lipoxygenase (LOX) and cyclooxygenase (COX) thereby reducing the level of pro-inflammatory factors (Chen et al., 2002). GPX4 reduces the release of pro-inflammatory mediators and inflammatory response associated with AS, which has been proved a chronic vascular inflammatory disease.

#### 4.1.3 NRF2 Increases the Production of NADPH

The production of GSH (GSH) and thioredoxin (TRX) eliminates peroxides and thus inhibits ferroptosis (Chen et al., 2002), while NADPH deficiency will lead to a decrease of GSH and TRX, promoting the accumulation of lipid ROS (Yang et al., 2020a). The mammalian TRX system is an important reductase system (Arnér, 2020). AS a system that scavenges harmful lipid peroxides, FSP1 was found to be primarily localized in lipid droplets and plasma membranes.

Further studies have shown that FSP1 reduces coenzyme Q10 via NADPH to inhibit ferroptosis (Zhu et al., 2020). The NADPH/FSP1/CoQ10 system is therefore a negative regulator of ferroptosis. Glucose produces NADPH via the pentose phosphate pathway (PPP), which positively regulates ferroptosis via key enzyme G6PD (Zheng and Conrad, 2020). NRF2 can regulate the expression of G6PD (Dodson et al., 2019) (Figure 2).

The TRX system has been shown to play an important role in the regulation of metabolic processes, insulin signaling, regulation of blood pressure and inflammation (Poznyak et al., 2020a). It has been shown that Trx2 protects intravascular homeostasis by balancing mitochondrial ROS production in endothelial cells (Kirsch et al., 2016). Down-regulation of Trx1 inhibits the expression of VCAM-1 and ICAM-1 and blocks the initiation of AS (Chen et al., 2013). TRX also modulates macrophage inflammation and polarization (Tinkov et al., 2018). Hypercholesterolemia causes the accumulation of lipid peroxides in the aorta of ApoE^−/−^ mice, and the antioxidant effect of coenzyme Q10 inhibits the development of AS (Witting et al., 2000; Bentzon et al., 2014). In addition to the pentose phosphate pathway (PPP), NADPH production also occur through NADH phosphorylation catalyzed by NAD kinase (NADK) and the NADP-dependent conversion of isocitrate to α-KG by isocitrate dehydrogenase (IDH) (Zheng and Conrad, 2020). NRF2 increases NADPH via G6PD, and NADPH acts as an antioxidant to cells by regulating GSH, TXN and CoQ10, which inhibits the development of AS.

### 4.2 p53

#### 4.2.1 p53 Promotes the Development of Ferroptosis in AS

p53 can inhibit the synthesis of GSH by SLC7All in a transcription-dependent manner (Jiang et al., 2015). p53 increases the expression of GLS2 (glutaminase2) to catalyzes the hydrolysis of glutamine, which increases cellular susceptibility to ferroptosis by downregulating cellular antioxidant through massive hydrolysis of GSH and increasing ROS levels (Gao et al., 2015). PTGS2 is a key enzyme in the initiation step of prostaglandin synthesis and regulates the sensitivity of cells to ferroptosis by regulating the levels of key intracellular membrane phospholipids PE. When ferroptosis inducers such as GLS2 and erastin were applied to p53 wild-type cells, the cells exhibited upregulated PTGS2 gene expression and ferroptosis, but when p53-deficient cells were induced with GLS2 and erastin, PTGS2 gene expression levels were unchanged and ferroptosis did not occur. PTGS2 has been used as a marker of ferroptosis (Yang et al., 2014).

Deletion of SAT1, target gene of p53, decreases cell death induced by ROS. SATl increases the expression of ALOX15 (arachidonate-15-lipoxygenase), an iron-dependent PUFA oxidase that increases lipid peroxidation and thus promotes ferroptosis (Ou et al., 2016). p53 also promotes cellular ferroptosis by activating ALOX12 (arachidonate-12-lipoxygenase) (Chu et al., 2019). 12/15-LOX promotes increased trans-endothelial transport of ox-LDL and deposition of ox-LDL in the sub-endothelial space (Li et al., 2018). ALOX12 exhibits higher methylation levels in atherosclerotic plaques, particularly in endothelial cells, which is essential for p53-mediated ferroptosis following stress (Ouyang et al., 2021). The p53 gene also promotes ferroptosis through histone modification with non-dependent of p53 (Dai et al., 2020). p53 can form a nuclear p53-USP7 protein complex with de-ubiquitin specific peptidase 7 (USP7), further reduces the level of histone H2B monoubiquitylation (H2Bub1, a histone modification) mediated expression of SLC7A11, ultimately leading to ferroptosis (Wang et al., 2019a).

MDM2 and MDMX were found to be negative regulators of p53 by regulating FSP1 expression (Venkatesh et al., 2020) and suggests altering the lipid distribution of cells to promote the development of ferroptosis (Venkatesh et al., 2020). In addition, excess iron activates ROS production and induces lipid peroxidation in macrophage-derived foam cells, leading to instability of atherosclerotic plaques. Disturbed iron metabolism in macrophages is involved in the inflammatory response that exacerbates the severity of AS, and iron overload also closely related to the induction of macrophage polarization towards the inflammatory phenotype M1 via the ROS/acetyl-p53 pathway (Zhou et al., 2018; Handa et al., 2019; Ouyang et al., 2021).

#### 4.2.2 p53 Inhibits the Onset of Ferroptosis in AS

p53 can also regulate cell cycle regulation to induce ferroptosis. p21 can inhibit ferroptosis by increasing intracellular GSH leading to increased synthesis of GPX4, while p53 directly regulates the activation of p21, although the exact mechanism is unclear (Tarangelo and Dixon, 2018; Tarangelo et al., 2018). Senescence and ROS production in human aortic endothelial cells are accompanied by significant increase in p53 and p21 (Iwabayashi et al., 2012). In addition, p21 also promote VSMC senescence (Moon et al., 2004; Kim and Moon, 2005; Wang et al., 2015). p53 inhibits ferroptosis by blocking dipeptidyl peptidase 4 (DPP4), a multifunctional serine protease involved in glucose control and a regulator of ferroptosis and lipid metabolism. DPP4 binds to nicotinamide adenine dinucleotide oxidase 1 (NOXl), leading to the production of ROS in cell membranes and plasma, resulting in a large accumulation of intracellular lipid peroxides and ferroptosis (Xie et al., 2017).

### 4.3 Hippo Pathway

**FIGURE 3:**
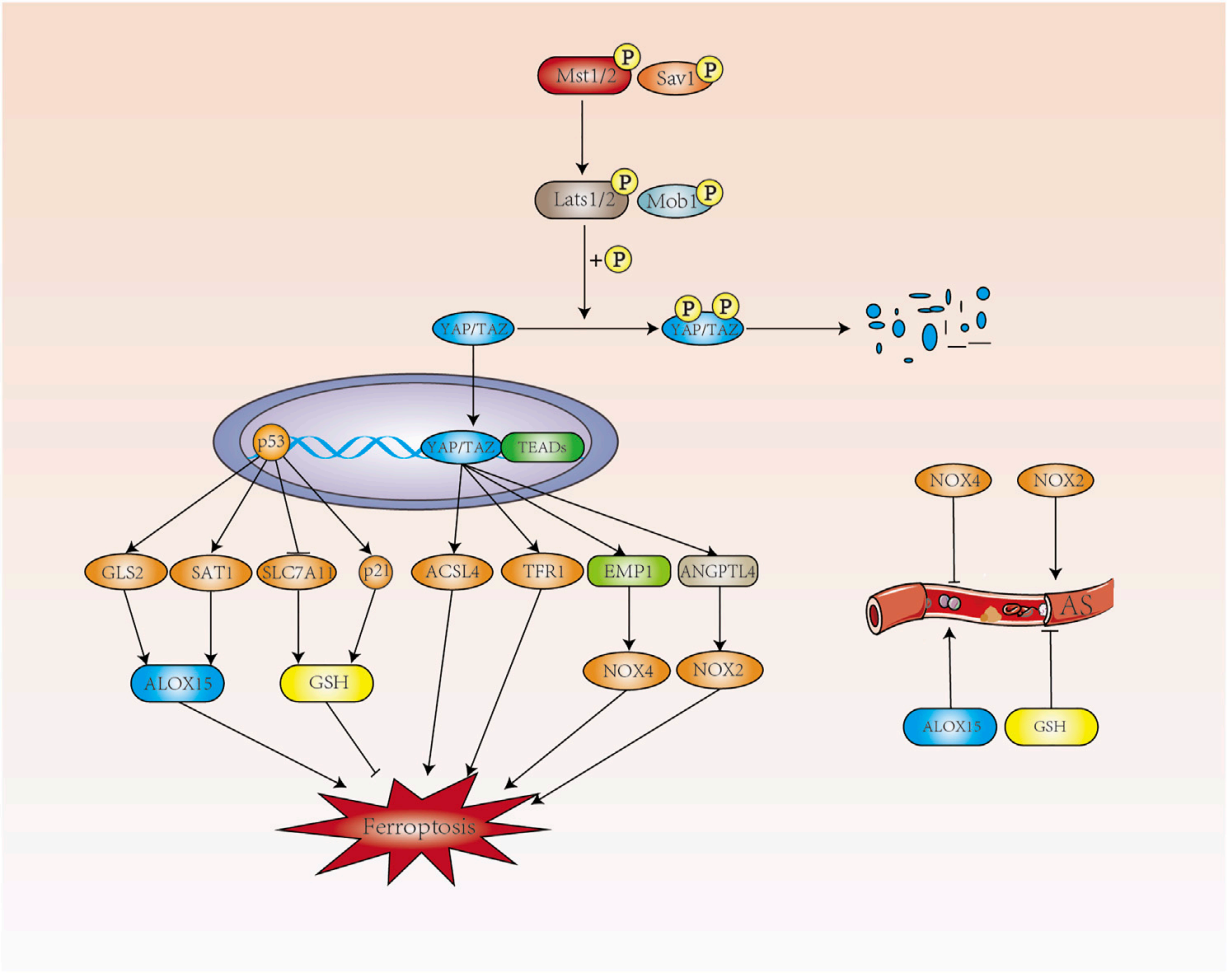
The p53 and Hippo pathways regulate ferroptosis. p53 promotes the expression of ALOX15 in a transcription-dependent manner to promote ferroptosis, and ALOX15 can contribute to the development of AS. p53 regulates ferroptosis by inhibiting GSH through SLC7A11 and promoting GSH production through p21. In contrast, GSH can inhibit the development of AS. When the Hippo signaling pathway is opened, MST1/2 phosphorylates MOB1 and LATS1/2 and increases the interaction between them. Phosphorylated YAP/TAZ by LATS1/2 is degraded in the cytoplasm with the assistance of SAV1. When the Hippo signaling pathway is closed, the level of YAP phosphorylation decreases and translocates into the nucleus where it binds to the transcription factor TEAD to produce ACSL4, which promotes intracellular lipid peroxidation. TFR1, which increases intracellular Fe^2+^ concentration, and NOX4 and NOX2, induce intracellular ROS production and development of ferroptosis.

The Hippo pathway consists of a series of protein kinase and transcription factors that is highly conserved from lower to higher animal. When the Hippo signaling pathway is shut down, the level of YAP phosphorylation is reduced and translocated into the nucleus, where it binds to the transcription factor TEAD, triggering the expression of a range of genes associated with cell proliferation. When the Hippo signaling pathway is turned on, MST1/2 phosphorylates and increases the interaction between MOB1 and LATS1/2 with the assistance of the co-factor SAV1 (Johnson and Halder, 2014). Hippo pathway plays an important role in cell growth, proliferation, metabolism and immunity, which has been a hot topic of research in the cardiovascular field (Figure 3).

The levels of AA (arachidonic acid) and AdA (adrenic acid) are closely related to the occurrence of ferroptosis. AA coenzyme A (AA-CoA) is produced by AA/AdA catalyzed by ACSL4 (acyl-coA synthetase long-chain family member 4) and eicosatetraenoic acid coenzyme A (AdA-CoA), both of which are key substrates for the onset of ferroptosis. Ferroptosis related with cell density in epithelial cells was mediated by E-calmodulin-mediated cell-cell contact, which activates Hippo signaling via the NF2 (also known as Merlin) tumor suppressor protein, thereby inhibiting the activity of the nuclear translocation and transcriptional co-regulator YAP, which can target including ACSL4 and TFR1 to promote ferroptosis (Wu et al., 2019a).

NADPH oxidase dysfunction plays an important role in the pathology of development of AS, including endothelial dysfunction, inflammation and vascular remodelling (Johnson and Halder, 2014). NADPH oxidase including NOX1, NOX2, NOX3 and NOX4, are the main sources of ROS during atherosclerotic formation (Poznyak et al., 2020b). In renal cell carcinoma, at low cell density, activated TAZ promotes the expression of epithelial membrane protein 1 (EMP1), followed by upregulation of NOX4 levels. NOX4 increases the level of intracellular lipid reactive oxygen species and induces ferroptosis (Yang et al., 2019). As a subtype of NADPH oxidase, NOX4 was shown to be AS-protective. Hydrogen peroxide released by NOX4 also inhibits the proliferation rate of vascular smooth muscle cells, prevents vascular remodeling and inflammation, and maintains the expression of eNOS under vascular stress (Schröder et al., 2012; Di Marco et al., 2016; Poznyak et al., 2020b).

In studying the role of TAZ in ferroptosis in ovarian cancer, angiopoietin like 4 was found to be a direct target gene regulated by TAZ, sensitizing ferroptosis through activation of NOX2 (Yang et al., 2020b). More importantly, ANGPTL4 maintains the integrity of the vascular endothelium by regulating vascular permeability, angiogenesis, inflammatory signaling, oxidative stress and lipid metabolism (Guo et al., 2014; Tsai et al., 2020). In addition, ANGPTL4 also regulates cellular energy homeostasis and reactive oxygen species and has been implicated in the pathogenesis of AS, including endothelial dysfunction, LDL oxidation and nitric oxide reduction (Zhu et al., 2012; Tsai et al., 2020). Hepatic ANGPTL4 deficiency significantly reduces triacylglycerol (TAG) and cholesterol levels, providing some protection against AS (Singh et al., 2021). When the Hippo pathway is closed, nuclear translocation of YAP induces endothelial cell proliferation and inflammation. YAP regulates the expression of ferroptosis-related target genes, including TFR1, ACSL4, EMP1 and ANGPTL4. Ferroptosis is promoted in terms of iron homeostasis, lipid metabolism and redox regulation, but whether ferroptosis is involved between Hippo and AS remains to be investigated.

## 5. Other Transcription Factors

### 5.1 ATF3 and ATF4

ATF3 was found to directly inhibit the expression of SLC7A11 in erastin-induced HT1080 cells, suggesting that ATF3 is a positive regulator of ferroptosis (Wang et al., 2020b). Endoplasmic reticulum stress promotes the upregulation of ATF3, which increases intracellular H_2_O_2_ levels through two pathways: ATF3 activates NOX4 transcription, overproduces of superoxide, inhibits SLC7A11 transcription, and deplets cysteine and GSH. H_2_O_2_ increases intracellular Fe^2+^ and restricts synthesis of GSH, leading to a continuous accumulation of toxic intracellular lipid peroxides (Lu et al., 2021).

Unlike ATF3, ATF4 plays a dual role in ferroptosis. Deletion of ATF4 increases ferroptosis in a variety of cancer cells (Chen et al., 2017; Zhu et al., 2017; Wang et al., 2019b). Inhibition of ATF4-HSPA5-GPX4 pathway-induced ferroptosis increases the anticancer activity of gemcitabine, which is related that ATF4 induces the binding of HSPA5 to GPX4, protecting against GPX4 protein degradation and subsequent lipid peroxidation (Zhu et al., 2017). Also, knockdown of SLC1A5 or CHAC1 or DDIT3 inhibited erastin or RSL3 induced ferroptosis (Dai et al., 2020). As the first cytoplasmic NADPH phosphatase, MESH1 leads to NADPH dephosphorylation, and the knockdown of MESH1 has been shown to increase NADPH and protect against ferroptosis (Ding et al., 2020). The knockdown of MESH1 induces different endoplasmic reticulum stress-related genes, particularly ATF3 gene. The knockdown of ATF4 sensitizes MESH1-deficient RCC4 cells to ferroptosis, suggesting a role in MESH1 knockdown-mediated protection against ferroptosis. The phenotypic similarity of stress tolerance caused by the removal of MESH1 and the accumulation of NADPH is achieved in part through the regulation of ferroptosis by ISR (Lin et al., 2021a).

In general, both ATF3 and ATF4 can regulate ferroptosis. ATF3 promotes ferroptosis by depleting intracellular GSH through inhibition of the system Xc-. Knockdown of ATF4 promotes ferroptosis, and protects against degradation of GPX4 through the ATF4-HSPA5-GPX4 pathway. In addition, endoplasmic reticulum stress promotes intracellular H_2_O_2_ levels by upregulating ATF3 and enhances intracellular Fe^2+^ by upregulating the expression of the transferritin receptor, leading to the accumulation of toxic lipid peroxides in cells. whether the high expression of ATF3 in AS is associated with ferroptosis needs to be further explored (Ouyang et al., 2021).

### 5.2 STAT3

Inhibition of STAT3 phosphorylation in endothelial cells down-regulates vascular endothelial growth factor (VEGF) expression, inhibits vascular endothelial cell proliferation and migration, and delays the formation of unstable plaques in AS (Dal Monte et al., 2011). STAT3 induces the proliferation and migration of vascular smooth muscle cells (Wang et al., 2016). STAT3 is involved in regulation of monocyte-to-macrophage differentiation, and inhibition of STAT3 activity decreases inflammation and monocyte-to-macrophage differentiation (Chen et al., 2019).

Protective effect of α6β4 integrin on erastin induced adherent epithelial cells and cancer cells is that α6β4-mediated activation of Src and STAT3 inhibits the expression of ACSL4 and suppresses lipid peroxidation (Brown et al., 2017). An earlier study showed that the onset of ferroptosis is not dependent on lysosomes, however, lysosomes play a potential role in promoting ferroptosis (Zhou et al., 2020). It has been shown that the transcriptional activity of NRF2 and STAT3 may jointly regulate ferroptosis-related gene expression, mainly because increased NRF2 can promote STAT3 phosphorylation, which amplifies downstream signaling and regulates SLC7A11 to inhibit ferroptosis (Liu and Wang, 2019; Qiang et al., 2020). The NF-κB/IL-6/STAT3 signaling pathway increases hepcidin expression and decreases serum iron and transferrin saturation (Yang et al., 2020a). Iron in macrophages is regulated by the hormone hepcidin, which reduces FPN-mediated iron export when iron is in sufficient supply. The expression of Hepcidin is associated with BMP signaling and its inhibition *in vivo* model has protective effect on AS, probably due to the fact that iron affects macrophage polarization and lipid metabolism levels (Wunderer et al., 2020).

Aberrant activation of STAT3 leads to endothelial cell dysfunction, macrophage polarization, inflammation and immunity, and thus may be an important regulator of the AS process (Chen et al., 2019). STAT3 regulates GSH synthesis by regulating SLC7A11 in ferroptosis and also inhibits ACSL4 expression, both of which can suppress the onset of ferroptosis. STAT3 also regulates intracellular iron homeostasis. How STAT3 regulates ferroptosis in AS needs to be further investigated.

## 6. Other Regulatory Molecules

### 6.1 SCD1 and FADS2

LSH (lymphoid-specific helicase) is a DNA methylation modifier that inhibits the progression of ferroptosis by directly upregulating lipid metabolism genes, including SCD1 (searoyl CoA desaturase) and FADS2 (fatty acid desaturase 2) (Jiang et al., 2017). SCD1 was found to have a rate-limiting effect in catalyzing monounsaturated fatty acid synthesis, which promotes tumor growth, migration and resistance to ferroptosis (Wang et al., 2016). SCD1 converts saturated fatty acids into monounsaturated fatty acids (MUFAs) to inhibit ferroptosis (Magtanong et al., 2019; Wang et al., 2020c). Highly active mutations in PI3K-AKT-mTOR signaling protect cancer cells from oxidative stress and ferroptosis through SREBP1/SCD1-mediated adipogenesis (Gao et al., 2021). Inhibition of SCD1 through activation of AMPK/SREBP1 to promote ferroptosis (Yi et al., 2020). Research has shown that lead decreases SCD and FADS2 expression, leads to a significant increase in MDA and ROS content in macrophages and may lead to foam cell formation and inflammation (Zhao et al., 2020). *In vitro* and *in vivo* results show that laminar flow protects endothelial cells by increasing endothelial cell SCD1 mRNA expression through a PPARγ-specific mechanism (Sun et al., 2015). Up-regulation of SCD-1 leads to desaturation of saturated fatty acids, promotes esterification and storage of saturated fatty acids, which protects HAECs from lipotoxic damage (Qin et al., 2007). The absence of SCD1 attenuates adipocyte inflammation and its paracrine regulation in macrophages and endothelial cells. Reduced oleic acid levels are associated with inflammatory regulation of SCD1 deficiency (Peter et al., 2008). Hypoxia-inducible factor (HIF) plays a crucial role in regulating the hypoxic response of tumor by altering cellular energy metabolism, including altering the expression of genes related to glucose and lipid metabolism. SCD1 and HIF-2a in human renal tubular epithelial cell lines promote tumorigenesis by maintaining cell survival, triggering cell migration and enhancing proliferation of cancer cells (Liu et al., 2010; Liu et al., 2010). The over-expression of SCD1 promotes lipid droplet (LD)-lysosome fusion through activation of TFEB nuclear translocation and its activity, which promotes lysosome biosynthesis and inhibits the formation of VSMC foam cell. Regulation of SCD1/TFEB mediated lipophagy may provide new therapeutic avenues for the treatment of AS (Stoyanoff et al., 2016).

LSH induces ELAVL1 (ELAV-like) through inactivation the expression of p53 RNA-binding protein. ELAVL1 enhances the expression level of LINC00336 by interacting with LINC00336 (Long noncoding RNA), increases the expression of CBS (cystathionine-β-synthase) and inhibits ferroptosis in lung cancer (Pi et al., 2019). DCAF8 and WDR76 were recently reported to act as substrate connectors and molecular inhibitors of the CRL4 (Cullin-4 RING ubiquitin ligase) system for the control of LSH stability. WDR76 competitively inhibits DCAF8-targeted LSH protein hydrolysis for lipid hydroperoxide-induced ferroptosis (Wang et al., 2019c). LINC00618 decreases the expression of SLC7A11 by interacting with LSH, thereby inhibiting ferroptosis. Furthermore, over-expression of LINC00618 increases the levels of Bax and cleaved caspase-3, promoting apoptosis. Importantly, LINC00618 was found to accelerate ferroptosis via apoptosis, but the potential interaction between ferroptosis and apoptosis remains poorly understood (Huang et al., 2021).

### 6.2 HSPs

The heat shock response (HSR) is a highly conserved stress response to maintain dynamic protein homeostasis in almost all eukaryotic cells (Wang et al., 2021a). As molecular chaperone, heat shock proteins (HSPs) are classified mainly on the basis of their molecular size into seven large families: Hsp110, Hsp100, Hsp90, Hsp70, Hsp60, Hsp40 and small HSPs (approximately 15–30 kDa) (Wang et al., 2021a). HSPB1 (also known as HSP25 in mice and HSP27 in humans) is a small heat shock protein and also HSP27 is an estrogen receptor beta (ER-β)-related protein (Miller et al., 2005; Barna et al., 2018). HSPB1 reduces cellular iron uptake by down-regulating TFR1 (Chiu et al., 2018). Protein kinase C-mediated phosphorylation of HSPB1 provides protection against ferroptosis by reducing iron-mediated production of lipid reactive oxygen species. Inhibition of the HSF1-HSPB1 pathway and HSPB1 phosphorylation increases erastin induced ferroptosis. This suggests that HSF1-HSPB1 is a negative regulator of ferroptosis (Chen et al., 2006).

HSP27 is a novel biomarker of AS that predicts adverse cardiovascular events (Sun et al., 2015). HSP27 prevents the formation of foam cell and plaques in female but not male mice (Inia and O’Brien, 2021). Intracellular HSPB1 stabilizes plaques by reducing vascular smooth muscle cell apoptosis (Rayner et al., 2009). Vascular wall infiltration of LDL may reduce VSMC adhesion and migration by inducing changes in HSP27/pHSP27 (Martin-Ventura et al., 2006). ROS were found to activate p38/MAPK phosphorylation, mediating the activation of HSF1, which in turn inhibits the development of ferroptosis (García-Arguinzonis et al., 2010). Another study showed that the lipid peroxidation metabolite 4-HNE could also mediate the activation of HSF1 through p38/MAPK phosphorylation, promoting the transcription of PROM2 (prominin2) and resisting the development of ferroptosis (Liu et al., 2021). Studies have shown that prominin2 promotes resistance to ferroptosis in mammary epithelial cells and breast cancer cells. Mechanistically, prominin2 promotes the formation of ferritin-containing multi-vesicular vesicles (MVBs) and exosomes transports iron out of cells (Brown et al., 2021). Oentin-1 inhibits VSMC migration by inactivating the NOX-ROS-p38-HSP27 pathway (Brown et al., 2019). PA (Palmitic acid) cause lipotoxic injury in cardiomyocytes, and over-expression of HSF1 restores the disturbance of iron homeostasis by mediating the expression of iron metabolism-related proteins such as TFRC, SLC40A1 and FTH1 (Lin et al., 2021b). Thus HSF1 appears to protect cardiomyocytes from PA induced ferroptosis by regulating the expression of iron-associated proteins and GPX4 (Wang et al., 2021b).

Heat shock protein A family (HSP70) member 5 (HSPA5, also known as BIP or GRP78) is a member of the HSP70 family and is mainly distributed in the endoplasmic reticulum (Zhang et al., 2010; Bailly and Waring, 2019). As mentioned above, ATF4 also induces the binding of HSPA5 to GPX4, thereby protecting GPX4 protein degradation and inhibiting ferroptosis. HSP70 facilitates over-expression of monocyte adhesion molecules and progression of AS (Xie et al., 2016). However, in parallel to the inhibition of HSP90 activity, a reduction in arterial wall inflammation and oxidative stress was also observed in association with increased HSP70 expression (Madrigal-Matute et al., 2010). HSP70 induces Treg to act as an anti-inflammatory agent (Wendling et al., 2000). When proteins carry a specific KFERQ-like motif to be degraded by CMA, GPX4 has a potential CMA-targeting motif that is recognized by HSPA8 and then transferred from the cytoplasm to the lysosomes via LAMP2A, ultimately causing protein degradation (Kaushik and Cuervo, 2018). HSP90-mediated LAMP2A protein stability promotes erastin induced ferroptosis by forming the HSPA8-LAMP2A-GPX4 protein complex to promote CMA-mediated GPX4 degradation (Wu et al., 2019b). LAMP2A and HSPA8-mediated CMA promote erastin induced GPX4 degradation via lysosomes, and HSP90 has been shown to increase the stability of LAMP2A protein, thereby linking autophagy and ferroptosis, known as chaperone-mediated autophagy (Liu et al., 2020).

Over-expression of HSP90 is associated with the characteristics of plaque instability. Inhibition of HSP90 reduces inflammatory responses and oxidative stress (Rodríguez-Iturbe and Johnson, 2018). HO-1 protein transcription is also regulated by HSF1 and NRF2 (Inouye et al., 2018; Rodríguez-Iturbe and Johnson, 2018). And HSF1 is also involved in nuclear translocation and stabilization of p53 (Zhang et al., 2021). Over-expression of HSP27 is protective against AS and is also a negative regulator of ferroptosis. Although HSP70 can inhibit ferroptosis via the ATF4-HSPA5-GPX4 pathway, the roles of HSP70 in AS are currently controversial (Bielecka-Dabrowa et al., 2009).

## 7. Conclusion

The pathogenesis of AS is complex and involves three major cell types including endothelial cells, smooth muscle cells and macrophages. The development of ferroptosis is associated with restricted GSH synthesis, disturbances in iron homeostasis, accumulation of lipid peroxides and fatty acid synthesis, which are also closely linked to the development of AS. In this review, we describe the role of ferroptosis signalling pathways and transcription factors and other regulatory molecules in the development of ferroptosis, with a focus on the linkage of these signalling pathways and transcription factors and other regulatory factors between ferroptosis and AS. The blood vessel is not just a simple anatomical organ, but one with complex functions. AS is the basis for many cardiovascular pathologies, including myocardial infarction and cerebral infarction, and ferroptosis also plays a very important role in many systemic cardiovascular diseases. Current research into ferroptosis in AS is still at a very early stage. What role does iron death play in the pathogenesis of atherosclerosis, and in the involvement of other organ lesions based on atherosclerosis. We hope that this review will provide new insights into the relationship between AS and ferroptosis.
